# Personalized Diagnosis and Therapy for Multiple Sclerosis

**DOI:** 10.3390/jpm12061017

**Published:** 2022-06-20

**Authors:** Cristina Ramo-Tello

**Affiliations:** Hospital Germans Trias i Pujol, Ctra Canyet, s/n, 08916 Badalona, Spain; cramot.germanstrias@gencat.cat

This Special Issue, entitled “Personalized Diagnosis and Therapy for Multiple Sclerosis” encompasses eight publications that we consider relevant, because their reading will help the clinician working regularly with people who suffer from multiple sclerosis (MS).

We all know that the primary interest of the neurologist treating a person with MS is minimizing his/her risk of having a MS relapse. Interestingly, the diagnosis and treatment varies greatly among clinicians and research in this field is very scarce. This issue offers a manuscript [1] with definitions of key concepts and a series of simple and practical statements with specific therapeutic recommendations, including an algorithm, seeking to standardize the care process for MS.

Currently there are up to 19 drugs approved to avoid the onset of relapses but there is no clear evidence to guide fundamental decisions such as what treatment should be chosen in the first place. This issue contributes a critical narrative review of the evidence on this still unresolved important issue, whether it is better to offer our patients Escalation Therapy or Early Intense Therapy [2] to prevent long-term outcomes.

One of the drawbacks of starting treatment with drugs of higher efficacy from the beginning is the less favorable side-effect profile. The search for biomarkers is essential to establish a personalized and safe medicine. Biomarker research conducted by some of our colleagues evaluating CD49d saturation under natalizumab treatment [3] has made it possible to make progress in this direction. CD49d saturation is a stable biomarker that can be used to optimize an individual’s dosing schedule and establish a safety range to personalize the treatment and thereby reduce the risk of progressive multifocal leukoencephalopathy.

From the clinical point of view, the interest in the cognitive assessment of patients with MS has been increasing over the years. It has gone from being an undervalued symptom to one considered essential that requires continuous monitoring. In this Special Issue we are especially interested in cognition, and experts on the subject explain predictors of cognitive impairment helpful to identify patients at risk [4] and also confirm the presence of cognitive impairment in patients with neuromyelitis optica spectrum disorder and its impact on health-related quality of life [5]. On the other hand, it aims to boost the field of machine learning for cognitive prognosis because most investigations on machine learning for MS prognosis were geared towards predicting physical deterioration, while cognitive deterioration, although prevalent and burdensome, remained largely overlooked [6].

Physical and mental health is not only in the hands of the professionals, since the patient is also the guardian of their own health. As professionals we must strive to explain to the patient the evidence that lifestyle behaviors are associated with quality of life [7], advise them on those that best suit them, and encourage them to adhere to these behaviors.

Finally, we wish to thank the coronavirus crisis for the thrust it has given to the adoption of teleconsultation. The results of the studies carried out demonstrate that longitudinal clinical monitoring using real-time audiovisual teleconsultation over the Internet is feasible and well-received by patients with MS and that such an approach can be a promising new care strategy not only in the short term but also in the long term [8].

We believe that the papers in this special issue reflect our interest in researching for people with MS rather than researching about MS.
